# Transformation of the Biological Paradigm in Bone Regeneration: An Integrative Review

**DOI:** 10.3390/jdb14010014

**Published:** 2026-03-11

**Authors:** Diyana Vladova

**Affiliations:** Department of Veterinary Anatomy, Histology and Embryology, Faculty of Veterinary Medicine, Trakia University, 6000 Stara Zagora, Bulgaria; diyana.vladova@trakia-uni.bg

**Keywords:** bone regeneration, osteogenesis, biofabrication, 3D bioprinting, bioinks, cell–matrix interactions, tissue engineering

## Abstract

Bone tissue is among the most commonly transplanted tissues worldwide. The treatment of critical-sized bone defects remains a significant challenge, as there is currently no universally accepted experimental model or therapeutic standard. Recent advances in fundamental cell biology are driving a paradigm shift in approaches to bone regeneration, highlighting the transformative potential of biofabrication technologies that integrate tissue engineering with personalized regenerative strategies. Three-dimensional (3D) bioprinting technology enables precise control over the architecture and spatial distribution of cellular and biologically active components, facilitating the creation of complex, personalized bone constructs. Central to this process are bioinks and biomaterials that mimic the extracellular matrix (ECM) and provide an optimal microenvironment for cellular function. Despite the substantial body of accumulated data, a comprehensive theoretical framework for functional bone biofabrication has not yet been fully established, emphasizing both the challenges and the innovative potential of the field. This integrative review synthesizes current knowledge on bone biology—from embryogenesis and cell–matrix interactions to molecular and neural regulation—and links it to the opportunities offered by biofabrication. Particular attention is given to bioinks as mediators between cell biology and engineering sciences, as well as to strategies for creating biomimetic ECM, optimizing scaffold design, and guiding future research toward clinically translatable bone regeneration.

## 1. Introduction—Paradigm Shift in Bone Regeneration

Bone is the second most commonly transplanted tissue in the world, with over two million bone graft procedures performed annually [1,2,3]. Despite years of clinical experience and technological advances, it has been found that the treatment of critical-sized bone defects remains a serious challenge, as there is still no universally accepted experimental model or therapeutic standard. The main requirements for bone substitutes include optimal geometry, adequate mechanical strength, biological tolerance and minimal risk of infection. The global shortage of donor organs and tissues further emphasises the need for innovative alternatives to allografts and autografts [4].

Advances in fundamental cell biology have revolutionised biofabrication technologies, laying the foundations for tissue engineering (TE) and shifting the focus from conventional to personalised regenerative approaches [4,5,6]. Based on [4,5,6], three-dimensional (3D) bioprinting is establishing itself as a leading technology in TE, as it provides precise control over the architecture of the constructed structures, the spatial distribution of cells and biologically active substances. This allows the creation of complex, personalized bone substitutes with a high degree of functional integration [7,8,9].

Control over the porosity and microarchitecture of scaffolds is key to successful cell adhesion, proliferation, and differentiation [7,10]. The selection of suitable biomaterials (bioinks) that mimic the properties of the extracellular matrix is critical for maintaining an optimal microenvironment and promoting tissue homeostasis [3,11]. Based on these data, diverse strategies for optimising natural and synthetic scaffolds contribute to the dynamic development of technologies in the field of bone tissue biology and regeneration.

The last decade has seen an exponential increase in the volume of data related to bioprinting and bone tissue regeneration. Our search of the PubMed and Scopus databases using the keywords of this review identified numerous publications, including review articles and monographs [1,4,5,12,13,14,15,16,17,18,19,20,21,22,23,24,25,26,27]. Despite the significant amount of empirical data accumulated, there is still no consensus theoretical model that integrates mechanical stability and biological activity within a unified biofabrication strategy [3,7,20]. This avalanche of information, although a valuable source of innovation, can lead to epistemological paralysis (neophobia) [28], while revolutionary changes in the understanding of bone as an organ signal a fundamental paradigm shift [28,29].

Based on the synthesis of the reviewed literature, it can be suggested that the existing biological paradigm is based on a model in which bone tissue is viewed as a collection of cells and matrix with predictable connections between them. In the contemporary context, however, there is a transition to a systemic, integrative understanding of bone as a dynamic and self-organising structure. Bioink occupies a central place in this epistemological transition. It can be viewed not only as a biomaterial tool, but also as a mediator between two biological paradigms, materialising cells into technologically feasible bone tissue constructs.

This review aims to analyse precisely this paradigmatic transition in the biological perception of bone tissue regeneration by discussing the two phenomenal planes—the fundamental biology of bone tissue and the biofabrication of its functional replica, defining bioink as a key element of the new integrative language between them.

## 2. Literature Search Strategy

The literature search strategy was developed to ensure a comprehensive and unbiased synthesis of the available scientific evidence on bone tissue regeneration and biofabrication.

Relevant literature was identified through systematic searches of the PubMed and Scopus databases using keywords including: bone regeneration, osteogenesis, biofabrication, 3D bioprinting, bioinks, cell–matrix interactions, and tissue engineering. The search encompassed publications in English, including both classical sources and recent studies spanning from 1991 to 2025, reflecting key scientific advances and technological innovations. The review also drew on established knowledge from classical textbooks, which provided a foundation for understanding bone biology and tissue engineering principles.

Both experimental and review articles, as well as monographs providing relevant information on bone tissue biology or biofabrication strategies, were considered for inclusion. Following the initial screening, 180 publications were critically analysed and selected for inclusion in this review.

This approach ensures that the review integrates both fundamental theoretical knowledge and the most recent empirical data, emphasizing the intersection of cell biology and engineering approaches in bone tissue regeneration.

## 3. Fundamental Insights into Osteogenesis: From Embryology to Cell–Matrix Interactions

### 3.1. Osteogenesis: Classical Embryology View

The embryonic development of the skeleton in mammals begins with the condensation of MSCs from the neural crest or mesoderm and occurs through two different processes: endochondral ossification and intramembranous ossification [30,31,32,33]. In the development of the skeleton, the bones of the craniofacial and appendicular skeleton have different mechanisms of embryonic development [34]. According to the authors, the former develop through intramembranous ossification, while the latter are formed through endochondral ossification.

Endochondral ossification is a key mechanism in bone development, involving chondrocyte differentiation, mineralisation of the extracellular cartilage matrix, vascularisation and subsequent bone formation [35]. Endochondral ossification begins with the transformation of mesenchyme into hyaline cartilage, which is later replaced by newly formed coarse-fibrous bone tissue [36,37]. During the embryonic period, coarse fibrous bone tissue originates from mesenchyme and precedes the development of lamellar bone in the replacement of cartilage tissue [36]. As described in [36], chondrocytes proliferate rapidly and secrete ECM, forming the cartilaginous model of the bone, together with the covering perichondrium. Chondrocytes located near the centre of the bone pattern undergo hypertrophy and begin to add type X collagen and fibronectin to the matrix they produce, which subsequently allows calcification. Calcification of the ECM leads to chondrocyte apoptosis, forming cavities in the cartilage template. Blood vessels invade the newly formed cavities, further expanding the spaces and growing together to form the medullary cavity. In addition, blood vessels transport osteogenic cells and trigger the transformation of the perichondrium into periosteum [36].

The cells on the inner surface of the periosteum differentiate into osteoblasts and secrete osteoid parallel to that of the existing matrix, thus forming layers commonly referred to as compact bone [38]. Subsequently, osteoblasts create a thickened area of compact bone in the diaphyseal part of the periosteum, where the primary centre of ossification is formed. While the bone replaces the cartilage in the diaphysis, it continues to proliferate at the ends of the bone, forming epiphyseal plates, which play a role in the longitudinal growth of the bone [39]. After birth, this process is repeated in the epiphyseal region, where the secondary centre of ossification is formed [35,40].

During intramembranous ossification, mesenchymal tissue undergoes direct differentiation into bone [37]. Evidence from [37] indicates that mesenchymal cells originating from the neural crest transform into osteoblasts, which organise themselves into ossification centres. They secrete osteoid, a non-mineralised collagen–proteoglycan matrix capable of binding calcium. Mineralisation of the osteoid leads to hardening of the matrix and incorporation of osteoblasts, which transform into osteocytes. Continued secretion of osteoid around the blood vessels forms the spongy bone, whose vessels later form the red bone marrow. Mesenchymal cells on the surface of the bone form the periosteum, whose inner cells differentiate into osteoblasts and secrete new osteoid, forming layers of compact (cortical) bone.

Endochondral ossification occurs with calcified cartilage nuclei, which serve as a stable support for the formation of new bone tissue. Intramembranous ossification, on the other hand, is initiated in the connective tissue, where bone is initially formed through a static and subsequently through a dynamic process of ossification [41,42]. According to Shapiro [30], bone tissue is formed through the differentiation of osteoprogenitor cells into mesenchymal osteoblasts, which synthesise bone tissue in a random orientation, or surface osteoblasts, which synthesise bone on surfaces in a well-oriented lamellar series.

The modelling of the bone matrix from initial tissue to eventual lamellar orientation is essential for the bone to develop its maximum strength [30]. According to Chaldakov [43], type I collagen is a protomeric molecule in the morphogenesis of fibrils, which, together with phosphorus and calcium located in bone tissue, determine its resistance to mechanical stress.

### 3.2. Cells and Extracellular Matrix: Interactions and Mechanisms

As an organ, bone plays a key role in controlling critical physiological mechanisms, including haematopoiesis, homeostasis and mineral storage [44]. Morphologically, bone tissue is composed of various cellular elements—progenitor (osteochondrogenic) cells, osteoblasts, osteocytes and osteoclasts [37]. Osteochondrogenic progenitor cells are mesenchymal stem cells (MSCs) that can differentiate into chondrocytes or osteoblasts [45]. The osteogenic differentiation of MSCs involves four stages [15,46] (Table 1).

Understanding the osteogenic differentiation of MSCs is crucial for translating biological principles into biofabrication strategies. Table 2 summarizes the main stages of MSC osteogenesis, highlighting key characteristics and processes.

During embryonic development, mesenchymal condensation can differentiate directly into bone through intramembranous ossification, forming flat bones, while endochondral ossification creates a temporary cartilaginous matrix that subsequently mineralises [47]. In mice, primary ossification of long bones begins around the fifteenth embryonic day (E15), and secondary ossification around the fifth postnatal day (P5), as described in [47].

During postnatal development, osteochondrogenic progenitor cells are retained in the endosteum and periosteum [43]. Osteoblasts originate from MSCs and play a key role in maintaining the balance between bone formation and resorption, including the regulation of osteoclasts [48]. During the embryonic period, osteoblasts secrete osteoid, a non-mineralised matrix that subsequently calcifies and forms bone tissue. MSCs, osteoblasts, and osteocytes sense and respond to mechanical and biochemical signals from the ECM, regulating their own differentiation and function [49]. Osteoclasts are multinucleated cells that participate in bone resorption by secreting hydrogen ions. The latter cause acidification and degradation of bones [50,51]. In this way, each of the cellular elements participates in the dynamic remodelling of bone through a coordinated process of deposition and resorption [52], which in classical sources is defined as “unity and clash of opposites” [36,53].

Osteocytes are the most numerous cells present in the bone structure, organizing its remodeling [37,54]. They are formed from osteoblasts retained in the osteoid. Osteocytes connect to each other and to the ECM through cytoplasmic processes. During the postnatal period, cell–cell and cell–ECM communication allows them to be mechanosensors of bone stress and deformation [37,54]. According to Ferretti et al. [41], stationary osteoblasts not only provide the first osteocytes during osteogenesis, but these cells also function as mechanosensors. This points to the fact that bone perceives mechanical loads from the early stages of osteogenesis.

From the perspective of classical cell biology, the bone tissue model has a dynamic cell–matrix environment [43]. Skeletal formation in vertebrates and the functions of bone and cartilage structures are regulated by the interaction between extracellular, cell surface and intracellular molecules [40]. Regardless of the different mechanisms of embryonic development, both endochondral and intramembranous bone share similar major regulatory transcription factors and downstream growth factors [34].

As already mentioned, despite numerous studies, there is no general scientific consensus resulting in functional biofabrication in which cells actively build and remodel the matrix [3,7]. In this context, we find it appropriate to emphasise the fact that in order to successfully transcribe the bone tissue biological model into an identical biomimetic print, we need to refocus and deepen our understanding of general biological principles in the context of biofabrication.

From a biological point of view, it has been firmly established that the bone matrix consists of organic and inorganic components, whose composition, according to Lin et al. [15], varies depending on age, gender, etc. The organic component is synthesized by osteoblasts prior to the mineralization process and consists mainly of type I collagen and non-collagenous proteins [55]. In addition to type I collagen, which accounts for 90% of the total collagen in the bone ECM, type III and V collagen are also present [43,55].

Non-collagenous proteins form the following groups: proteoglycans (biglycan, decorin, keratocan, asporin), glycoproteins (osteonectin, thrombospondins, r-spondins), γ-carboxyglutamate-containing proteins (osteocalcin, matrix Gla protein (MGP), periostin) and small N-linked glycoproteins binding integrin (BSP, OPN, DMP1, MEPE) [46]. The inorganic component of the matrix mainly contains apatite (hydroxyapatite (HA) [46]. The collagen that is released during tissue mineralisation acts as a matrix for HA deposition [56].

### 3.3. Molecular and Neural Regulation of Bone Regeneration

Bone tissue is a dynamic and constantly remodelling structure whose normal functioning depends on complex cellular and molecular interactions [43]. The main cells involved in this process are osteoclasts, osteoblasts and osteocytes. In recent years, osteocytes have attracted increasing attention due to their key role in regulating bone metabolism through the release of sclerostin (SOST), a protein that suppresses osteosynthetic activity [57]. As described in [57], bone metabolism is controlled by both local and systemic factors, including neurotransmitters such as brain-derived serotonin, which stimulates the proliferation and differentiation of osteoblasts through activation of the Wnt/β-catenin signalling pathway. Disruptions in these regulatory mechanisms can lead to various skeletal pathologies, such as osteoporosis or high bone mass syndrome. In addition, the rich innervation of bone tissue provides a complex regulatory network of growth factors and hormones that help maintain bone homeostasis and regeneration [57].

Endochondral osteogenesis is a complex and tightly regulated process that involves coordinated interaction between multiple signaling pathways, transcription factors, and extracellular signals [31,32,33]. The initial stages of chondrogenesis are determined by Sox9, SHH, IHH, and PTHrP, which stimulate chondrocyte proliferation and cartilage template formation. The next stage involves intensive chondrocyte proliferation, controlled by FGF, TGF-β, and Notch, which ensures the growth and stability of the cartilage plate [31,37].

During chondrocyte hypertrophy, IHH, BMPs, and Wnt/β-catenin are activated, which prepare the extracellular matrix for mineralisation and stimulate the vascularisation necessary for subsequent osteogenesis [32,33]. Mineralisation and bone tissue formation are regulated by BMPs, Runx2, Osx, SOST and Wnt signalling, which initiate osteoblast differentiation, the formation of the bone matrix, and control the processes of mineralisation [31,33,37]. Bone metabolism is influenced by both local and systemic factors, including neurotransmitters such as brain-derived serotonin, which stimulates osteoblast proliferation and differentiation by activating the Wnt/β-catenin signalling pathway [57].

Osteocytes, traditionally considered key cells in bone remodelling, are also emerging as important mediators of bone regeneration. They release biochemical signalling molecules such as prostaglandins, nitric oxide, Wnt ligands, and IGF-1, which stimulate osteogenesis by activating mesenchymal stem cells [58]. Through interaction with MSCs and the extracellular matrix, osteocytes assist in the coordinated regulation of cell activity and tissue repair. The regulation of bone remodelling through RANKL, OPG, TGF-β and PTH ensures a balance between osteogenesis and resorption, maintaining bone tissue homeostasis [33,48,59,60].

The significant therapeutic potential of human multipotent mesenchymal stromal cells has long been recognised [61]. This potential is also highlighted in the studies by Uder et al. [62], who define human MSCs by a specific panel of cell surface markers (CD105^+^, CD73^+^, CD90^+^, CD34^−^, CD14^−^/CD11b^−^, CD79^−^/CD19^−^, HLA-DR^−^). Furthermore, their capacity for multiple differentiation—osteogenic, adipogenic and chondrogenic—is a key criterion for defining MSCs.

Bone marrow MSCs are often considered the reference standard for osteogenesis studies; however, MSCs from alternative tissue sources may exhibit different functional properties influenced by their origin and culture environment. For example, in studies by Saxer et al. [63], the stromal vascular fraction (SVF) from adipose tissue demonstrated the ability to promote bone fracture repair in nude rats through the formation of vascular and bone structures. These data highlight the potential clinical applicability of alternative sources of stromal cells in the treatment of bone defects and draw attention to the possibility of combined approaches in tissue engineering.

Although MSCs have multilinear potential, their actual contribution to tissue repair is mainly due to the secretion of endogenous trophic factors that attract host cells, promote local vascularisation and regulate the inflammatory response [64,65]. The extracellular matrix also plays a central role in regulating cell functions such as survival, migration, proliferation, and differentiation. As a dynamic structure, the ECM provides orienting signals for cells and modulates their gene expression, thereby supporting the normal development of endochondral bone [23,66].

In addition to classical mechanisms, neuroregulation plays an essential role in postnatal osteogenesis. Neuropeptides such as SP (substance P), CGRP and serotonin stimulate the proliferation and differentiation of mesenchymal stem cells, contributing to vascularisation and local growth of the bone matrix [39,58,67,68]. In the context of tissue engineering, this knowledge allows the development of biomaterials and scaffolds that deliver specific factors or create a microenvironment that promotes osteogenic differentiation and integration with host tissues (Table 3). These approaches form the basis of neuro-bone tissue engineering and personalized bone biofabrication. Future strategies in bone tissue biofabrication show promising prospects for revolutionary changes in the treatment of tissue defects.

The creation of 3D bioprinted scaffolds for tissue regeneration allows precise control over porosity, mechanical properties, and local delivery of growth factors [69,70,71]. The possibility of spatially and temporally controlled release of BMPs, TGF-β or SP provides a strategy for directing MSCs towards osteogenic differentiation while maintaining cell survival and functionality [72,73,74]. The rich innervation of bone tissue further provides a complex network of growth factors and hormones that influence bone homeostasis and regeneration [57].

Although interest in the regulatory role of the skeletal nervous system has only grown in recent years, the influence of sensory, sympathetic, and parasympathetic nerves on bone tissue has been studied for decades [75,76,77,78]. Information on the role of the peripheral nervous system (PNS) in bone regeneration remains limited, despite numerous studies [68]. After bone trauma, molecular and cellular changes are observed in neurons, peripheral nerve cells and the site of injury [68], which triggers bone regeneration. Clinical data show accelerated fracture healing in patients with traumatic brain injury, highlighting the role of neuroregulation [39]. As described in [39] an interesting phenomenon associated with postnatal neuroregulation of post-traumatic bone tissue regeneration is that patients with fractures and concomitant traumatic brain injuries demonstrate increased callus formation and faster fracture healing. This suggests that brain injury affects multiple factors in the healing process, including the release of inflammatory mediators, serum leptin concentration, and the differentiation potential of mesenchymal stem cells [39,79,80,81]. Emerging data suggest that various neural circuits and neuropeptides exert regulatory influences, deepening our understanding of the role of the central nervous system in bone metabolism. The PNS influences bone repair through neuropeptides, neurotransmitters, and interaction with stem cells (MSCs) that can differentiate into chondrocytes and osteocytes or dedifferentiate after injury [82,83]. Xie et al. [82] highlight the connection between the nervous and skeletal systems through the involvement of MSCs. Neuropeptides and neurotransmitters can also be produced by osteoblasts, osteocytes, and other non-neuronal cells, which requires careful interpretation of the function of the PNS [84,85,86,87,88].

Understanding the interaction between peripheral nerves and bone is important for developing therapeutic strategies to promote bone regeneration. Peripheral nerves typically follow blood vessels during both embryonic and postnatal development [89]. The process of bone regeneration is nerve-dependent, and neurotransmitters, neuropeptides, and the redifferentiation of nerve cells are key mechanisms [68].

Fracture healing proceeds through reactive, reparative, and remodelling phases, all of which are strictly regulated by the nervous system [90,91,92,93,94]. According to the authors, in tissue engineering, the implantation of a sensory or vascular nerve bundle can stimulate osteogenesis, possibly through the regulatory function of neuropeptides.

The integration of cellular mechanisms of osteogenesis, signalling pathways, neuro-regulation, and advanced biomaterials through 3D bioprinting and scaffold technologies has been highlighted in the literature as a promising approach for developing personalized therapies for bone defects.

## 4. Complexities and Solutions in Bone Biofabrication: Bioink as a Mediator Between Cell Biology and Functional Structure

Bone repair follows the same biological principles that determine its development during embryogenesis, but the specific mechanism of regeneration is largely determined by the provided biomechanical and cellular microenvironment [30]. However, the regenerative capabilities of bone tissue are limited, so that large defects cannot be spontaneously self-corrected [95,96]. A variety of studies show that TE plays an important role in the process of promoting cell migration, proliferation and differentiation [97,98,99,100,101]. However, current TE methods have limitations that can be significantly overcome with 3D bioprinting technology, including in the study/treatment of a number of diseases [8,102,103,104,105,106,107].

About 20 years ago, the interdisciplinary approach [108,109,110] of 3D bioprinting revolutionised medical technology and was introduced as one of the best solutions for tissue engineering, unattainable by conventional tissue bioproduction approaches [111]. Three-dimensional bioprinting has progressively developed into an unrivalled technology [112], allowing precise control over multiple compositions, spatial distributions, and architectural accuracy/complexity, thereby achieving effective recapitulation of the microstructure, architecture, mechanical properties, and biological functions of target tissues and/or organs [113,114,115,116,117]. In this context, scientists are pursuing multiple approaches based on the use of natural and/or artificial scaffolds, decellularised extracellular matrices (dECM) and, more recently, bioprinting. Scaffolds of increasing complexity are being produced, loaded or unloaded with differentiated or stem cells [110,118]. However, it is necessary to find an appropriate balance between mechanical and biological properties to achieve biomimetic tissue [119].

**Figure 1:**
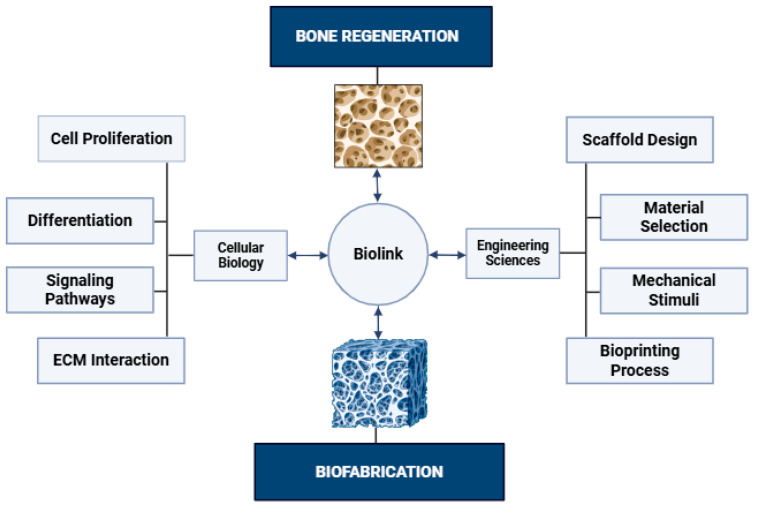
Conceptual framework illustrating bioink as a mediator between cellular biology and engineering sciences in bone biofabrication. Bioinks integrate biological components with engineering design principles to enable functional bone tissue construction. Created in Biorender. Diyana Vladova. (2026) https://app.biorender.com/illustrations/6910d7c8a20c981109166208?slideId=a359095d-41df-4d42-a8cb-42e77523b6ec (accessed on 2 February 2026).

An important part of the prototyping process is the selection and preparation of biomaterials, as they perform the function of the ECM (Figure 1).

Undoubtedly, scaffolds are three-dimensional biocompatible structures that can mimic the properties of tissue ECM, such as mechanical support and bioactivity, which provides a platform for cell adhesion, proliferation and differentiation, and promote constructive tissue regeneration [120,121,122,123,124]. The matrix material for tissue printing, or scaffold printing, often described as bioink, is central to this process and must provide appropriate cues and signals for cell function and tissue formation [125], including bone regeneration [126]. Numerous materials have been developed as such for/or scaffolds for applications in TE [127,128,129,130,131,132]. The evolution that bioprinting can bring to the field of scaffolds is enormous because it allows greater control over the architecture of the scaffold, especially its porosity [133,134]. Much research has been inspired by the control over scaffold porosity offered by 3D printing [135,136,137], as well as by the challenge of selecting a suitable bioink, the need for different types of improved bioinks and their evaluation [138,139,140,141,142,143,144,145]. According to some authors [146,147], the formulation of the new bioink must balance cell-supporting properties, printability, and mechanical properties that attempt to match the microenvironment of the target tissue.

Biomaterials for guiding the osteogenic differentiation of MSCs are a key component in tissue engineering. Leach et al. [64] emphasise that the biophysical properties of biomaterials—composition, mechanical characteristics, porosity and topography—can dictate the fate of MSCs. Despite the potential of growth factors, limitations such as the high doses required to achieve the desired effect and the difficulties in controlling their release motivate the development of new material-based approaches. The porosity of the scaffold is a critical parameter, with the optimal pore size being between 50 and 100 μm to promote mineralisation and cell invasion [64,148,149].

In this context, bone tissue biofabrication offers the possibility of reconstructing the complex three-dimensional microarchitecture of bone by using bioinks that combine cellular and matrix components in a spatially controlled model [3,7,10]. Bioinks are a key structural element of 3D bioprinting—their biocompatibility, rheological properties and structural stability—determine the success of the resulting tissue [26]. They can be natural (collagen, gelatin, fibrin, chondroitin sulphate, hyaluronic acid), synthetic (polyethylene glycol, polycaprolactone, polyvinyl alcohol) or hybrid, combining the mechanical resistance of synthetic and the biological activity of natural polymers [3,11,12,25,64,148,149,150,151,152,153,154,155,156,157,158,159].

Based on our earlier findings [70], collagen-based bioinks are particularly suitable for bone biofabrication, as they resemble the natural ECM and create an optimal environment for the osteogenic differentiation of MSCs [23,24,81]. In bone tissue, type I collagen is the most abundant protein and the most commonly used form of collagen in bioprinted bone scaffolds [160]. Collagen provides low immunogenicity, permeability, good biocompatibility and biodegradability, and has the potential to regulate cell morphology, adhesion, migration, and differentiation of cells [161,162], although its slow gelation kinetics and low viscosity may limit its bioprintability [163]. The combination of type I collagen with hyaluronic acid (HA) results in osteochondral scaffolds with ECM suitable for osteoblasts [164].

Critical parameters in the development of bioinks are viscosity, gelation rate, cell viability, and structural stability after printing [165]. Effective osteogenesis requires a material that maintains high cell density and provides sufficient mechanical strength for mineral deposition [109]. Additionally, the inclusion of oxygen-generating biomaterials [166] and the co-cultivation of stem and progenitor cells with primary cells enhances tissue formation efficiency through paracrine factors and the attraction of endogenous cells [167,168]. The addition of bioactive nanoparticles such as hydroxyapatite or bioglass stimulates mineralisation and osteoconductive properties [159,169,170]. “Smart” bioinks, sensitive to temperature, pH and mechanical stress, allow dynamic control of cell differentiation and tissue maturation [16]. Hydrogel matrices integrate growth factors such as BMP-2, VEGF and TGF-β1, mimicking the natural bone microenvironment [68,79] (Table 4).

However, most of the matrix materials used for bioprinting to date cannot represent the complexity of the natural ECM, i.e., they cannot restore the inherent cell morphofunctionality [171,172]. Not all criteria for an ideal scaffold have been met yet [173], despite active scientific research in the field of development/production of highly effective scaffolds, including biomaterials. Against this background, it is clear that existing bone tissue biomimetic models still do not fully reflect the dynamic cell–matrix organisation outlined by the classical paradigm of biology [43,174], which requires a rethinking and upgrading of approaches in tissue engineering [20]. In line with the findings reported in [20], contemporary strategies are focused on integration between cell biology and engineering technologies. Osteogenesis and bone tissue remodelling are achieved through close interaction between MSCs, ECM and regulatory biomolecules that coordinate cell communication and tissue organization [43].

A key challenge remains the creation of scaffolds that meet the structural, mechanical, and osteoconductive requirements of natural bone and support vascularisation [20]. 3D scaffolds provide a temporary environment for ECM, cell activity, oxygen diffusion, nutrient delivery and waste removal, while providing mechanical support and gradually remodelling [175].

Despite advances, biofabrication of bone tissue faces challenges related to long-term cell viability, controlled vascularisation, and in vivo integration [3,11,176]. The synthesis and maintenance of bone tissue are highly dependent on adequate blood supply and active intercellular communication via the lacunar–canalicular system [27,30]. Contemporary research highlights the complexity of these processes, emphasizing the molecular cascades regulating cell differentiation, the role of structural proteins—especially the different types of collagen—and the importance of tissue vascularization for successful recovery. Yang et al. [27] critically evaluate the composition of biomaterials that should promote osteogenic–angiogenic coupling by modulating key signalling pathways.

In addition, biophysical signals influence the survival and differentiation of MSCs, with osteogenically induced cells showing higher survival and engraftment in vivo [177], but it remains unclear whether this is due to the biophysical properties of the materials or to the targeting of cell fate. The lack of standardized protocols limits comparability between research and clinical implementation [26].

4D biofabrication technology uses stimulus-responsive biomaterials for dynamic scaffolds that can change their shape, size, and properties over time to mimic bone development and regenerative processes [178,179,180]. This allows for more precise modelling of osteogenesis, tissue maturation and interaction with the microenvironment. In addition, the approach avoids the need to create vessel-like networks in scaffolds due to the use of self-folding tubes [178]. According to the same study, optimisation of bioink from stimulus-responsive biomaterials must also consider printability and cell viability, which can represent an additional challenge.

Future trends focus on the development of “smart” bioinks that integrate biological signals, nanostructured fillers and controlled release systems for osteoinductive factors, as well as the use of bioreactors to simulate physiological conditions [4,16,109]. Based on these observations, it can be envisaged that the introduction of artificial intelligence to optimise the composition and printability of biomaterials in real time could accelerate the transition from laboratory prototype to clinically applicable bone constructs.

## 5. Conclusions

At a conceptual level, the biofabrication of bone tissue represents not just a technological innovation, but a profound transformation of the scientific paradigm. It shifts the biological perception of bone as an organ towards a dynamic, self-regulating biosystem, amenable to engineering modelling and synthetic reproduction. In this sense, bioinks act as a mediator between cell biology and engineering sciences, forming a new integrative language of tissue regeneration. As Chaldakov [28] and Kuhn [29] note, scientific revolutions occur when the accumulation of facts causes a change in the way we think about the nature of the object. In the case of bone biofabrication, this change is already underway, driven by the quest to create functional, vascularised and integrated bone tissue. Future research will focus on the optimisation of bioinks, the design of complex 3D structures and the integration of cellular, biochemical and biophysical signals in order to achieve personalised and effective strategies for bone tissue regeneration.

## Figures and Tables

**Table 1:** Stages of osteogenic differentiation of mesenchymal stem cells (MSCs).

Stage	Description	Key Characteristics/Processes	Source
Adhesion	MSCs adhere to the osteogenic line and form osteoprogenitor cells.	Determination of cell fate; expression of osteogenic markers; activation of signals such as BMP, Wnt and Runx2.	[15,46]
Proliferative phase	Osteoprogenitors divide intensively. They express genes related to the cell cycle and histone signals.	Expression of genes related to the cell cycle and histone signals; increase in cell population.	[15,46]
Secretion of extracellular matrix (ECM)	Immature osteoblasts begin to secrete matrix proteins.	Formation of type I collagen and other ECM components; morphological changes; preparation of the matrix for mineralization.	[15,46]
Mineralisation	Mature osteoblasts mineralise the osteoid and transform into osteocytes.	Deposition of calcium phosphate crystals; terminal differentiation of osteoblasts; formation of bone tissue.	[15,46]

**Table 2:** Stages of MSC Osteogenic Differentiation: In vivo vs. In vitro.

Stage	In Vivo	In Vitro
Adhesion	MSCs adhere to the natural ECM and mechanical signals; cell fate determination.	MSCs adhere to collagen-coated plates and respond to added growth factors.
Prolife ration	Expansion regulated by niche factors and mechanical signals.	Supported by culture factors; absence of natural mechanical stimuli.
ECM secretion	Organized ECM preparing for mineralization.	Partial or uneven ECM; scaffold or bioink provides structural support.
Mineralisation	Mineralization occurs in a 3D environment with physiological ions; formation of functional bone.	Mineralization may be incomplete or unstructured; scaffold and inducing factors assist the process

**Table 3:** Signaling pathways and key factors in endochondral osteogenesis and their connection to biomaterials and tissue engineering.

Osteogenesis Stage	Key Factors/Signaling Pathways	Function/ Effect	References/Authors	Connection to Biomaterials/ Tissue Engineering
Chondrogenesis (early)	Sox9, SHH, IHH, PTHrP	Stimulates chondrogenesis, supports chondrocyte proliferation	[31,32,33]	Biomaterials can deliver growth factors and signals to direct MSCs toward chondrogenic differentiation.
Chondrocyte proliferation	FGF, TGF-β, Notch	Controls cell cycle, regulates growth of the growth plate	[31,37]	Scaffolds with optimized porosity and mechanical properties support cell proliferation and growth.
Chondrocyte hypertrophy	IHH, BMPs, Wnt/β-catenin, VEGF	Initiates hypertrophy, prepares ECM for mineralization, stimulates vascularization	[32,33,57]	BMPs and other osteoinductive factors in biomaterials can promote osteogenic differentiation and vascularization.
Mineralization and osteogenesis	BMPs, Runx2, Osx, SOST, Wnt	Osteoblast differentiation, bone matrix formation, regulation of mineralization	[31,33,37]	Bioinks and hydrogels can provide localized controlled release of BMPs and Wnt to stimulate bone matrix formation.
Bone remodeling regulation	RANKL, OPG, TGF-β, PTH	Balances osteogenesis and resorption	[33,59,60]	ECM-like biomaterials can provide signals to osteoblasts and osteoclasts, supporting remodeling.
Neuroregulation of osteogenesis	SP (substance P), CGRP, serotonin, neuropeptides	Stimulates MSC proliferation, osteoblast differentiation, vascularization	[38,39,58,67,68]	Implantation of scaffolds with neuroactive components or SP can promote osteogenesis; summary of neuro-bone engineering concepts.

**Table 4:** Representative bioinks and biomaterial systems used in bone tissue biofabrication, highlighting their main biological and mechanical characteristics.

Type of Bioink/ Material	Composition/ Additives	Key Advantages	Challenges/ Limitations	References
Natural polymers	Collagen type I, gelatin, fibrin, HA	High biocompatibility, supports MSC differentiation	Low viscosity, slow gelation	[81,163]
Synthetic polymers	PEG, PCL, PVA	High mechanical strength, tunable properties	Poor cell interaction	[25,152]
Hybrid bioinks	Collagen + HA, Collagen + PCL	Combines mechanical and biological advantages	Requires optimization of ratios	[164,169]
Bioactive composites	Hydroxyapatite, bioactive glass nanoparticles	Enhances mineralization and osteoconductivity	Risk of brittleness	[159,170]
Smart hydrogels	pH- or thermo-sensitive materials	Dynamic cell differentiation control	Complex fabrication	[16,79]

## Data Availability

No new data were created or analyzed in this study.
